# Droplet Impact on Surfaces with Asymmetric Microscopic Features

**DOI:** 10.1021/acs.langmuir.1c01813

**Published:** 2021-09-01

**Authors:** Susumu Yada, Blandine Allais, Wouter van der Wijngaart, Fredrik Lundell, Gustav Amberg, Shervin Bagheri

**Affiliations:** † Department of Engineering Mechanics, Royal Institute of Technology, 100 44 Stockholm, Sweden; ‡ École Normale Supérieure de Lyon, 69342 Lyon, France; § Division of Micro and Nanosystems, Royal Institute of Technology, 100 44 Stockholm, Sweden; ∥ Södertörn University, 141 89 Stockholm, Sweden

## Abstract

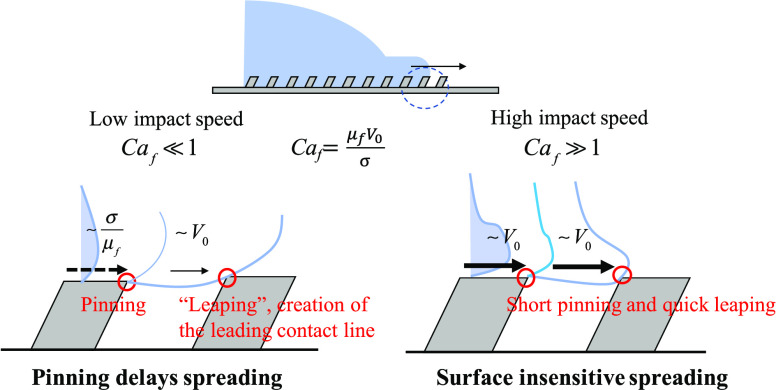

The impact of liquid drops on a rigid surface is central in cleaning, cooling, and coating processes in both nature and industrial applications. However, it is not clear how details of pores, roughness, and texture on the solid surface influence the initial stages of the impact dynamics. Here, we experimentally study drops impacting at low velocities onto surfaces textured with asymmetric (tilted) ridges. We found that the difference between impact velocity and the capillary speed on a solid surface is a key factor of spreading asymmetry, where the capillary speed is determined by the friction at a moving three-phase contact line. The line-friction capillary number *Ca*_f_ = μ_f_*V*_0_/σ (where μ_f_,*V*_0_, and σ are the line friction, impact velocity, and surface tension, respectively) is defined as a measure of the importance of the topology of surface textures for the dynamics of droplet impact. We show that when *Ca*_f_ ≪ 1, the droplet impact is asymmetric; the contact line speed in the direction against the inclination of the ridges is set by line friction, whereas in the direction with inclination, the contact line is pinned at acute corners of the ridges. When *Ca*_f_ ≫ 1, the geometric details of nonsmooth surfaces play little role.

## Introduction

The impact of droplets on a solid surface is essential in technological applications such as spray coating and cooling,^1,2^ pesticide deposition,^3,4^ and inkjet printing.^5,6^ The complex fluid–surface interaction during the impact—which includes splashing^7−11^ and trapping of a thin gas film underneath the droplet^12−15^—has been studied theoretically,^16−19^ numerically,^18−21^ and experimentally.^22−28^ These studies have established useful scaling laws of maximal deformation, which among other things are reviewed in ref (2, 29).

The influence of surface roughness and microstructures on drop impact has also been studied extensively focusing on different aspects, such as splashing,^23,29−31^ bouncing,^32−35^ trapped gas film under the droplet,^14^ rolling speed after the impact on inclined substrates,^36^ and maximum spreading radius.^25,26,37,38^ These studies have reported that surface topology influences the spreading and even small roughness delays spreading at a low impact velocity.^25^ However, it is not completely understood which microscopic features of a complex surface texture have the largest influence on droplet impact.

One example of a complex surface is an asymmetric textured surface, i.e., where the unit structure (post, ridge, rising, etc.) is not symmetric to the vertical line passing through the center of the structure. Asymmetric surface textures are used by natural organisms to control approaching raindrops.^4^ For example, the slanted microgrooves on the peristome of the “pitcher plant” *Nepenthes alata*(39−41) do not only assist to maintain the surface wetted, but they also prevent drops from falling into the pitcher tank.^42^ Although these asymmetric surface structures have been mimicked for technical applications such as oil–water separation^43^ and raindrop shielding,^42^ their influence on droplet impact is not fully understood.

Here, we perform droplet impact experiments on surfaces with asymmetric microstructures. We measure the spreading radius in different surface-parallel directions and quantify the droplet asymmetry by introducing a line-friction capillary number *Ca*_f_ = μ_f_*V*_0_/σ, where *V*_0_ and σ are the impact velocity and surface tension, respectively, and μ_f_ is the local friction at the moving vapor/liquid/solid phase contact line. As μ_f_ constitutes the key ingredient in our analysis (in contrast to earlier models^17,18,26^), we first briefly summarize the notion of contact-line friction, before discussing the scope of the present study.

### Contact-Line Friction

When a moving contact line exhibits a dynamic contact angle different from the static value, we expect a local dissipation at the contact line. de Gennes^44^ (eq. 4.71, p 860) introduced a local dissipation proportional to μ_f_*U*^2^ near the moving contact line, where *U* is the contact line speed and μ_f_ is a “simple friction coefficient” with the same dimensions as viscosity (denoted η_l_ in de Gennes’ original paper). This dissipation is expected from fundamental principles of thermodynamics, and it can have different molecular or hydrodynamic origins. Assuming a microscopic cutoff region where fluid slip is allowed,^45^ the dissipation due to slip and viscous friction in the vicinity of the contact line can be viewed as a local dissipation. Under different circumstances, the moving contact line can be treated as a thermally activated process, which is the basis for the molecular kinetic theory (MKT).^46,47^ See the recent reviews^48,49^ for discussions of these and other possibilities.

Regardless of its molecular origin, the parameter μ_f_ can be treated as a macroscopically relevant parameter that characterizes the contribution to the total dissipation from processes that are local to the contact line region. As such, it is expected to depend on the combination of the liquid and the substrate properties, as well as on the local dynamic contact angle, but not otherwise on the macroscopic flow geometry such as droplet radius or the length scale of surface geometry. Equivalent parameters have been introduced and used in the literature, for instance, as a linearization of an assumed smooth dependence of contact line speed on dynamic contact angle.^50^ Yue and Feng discussed contact line dissipation in the Cahn–Hilliard model and derived the resulting relation between contact line speed and the dynamic contact angle. Their relation, in our notation, is^51^
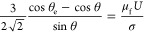
1where θ_e_ is the static contact angle, θ is the dynamic contact angle, and σ is the surface tension.

The contact line friction coefficient can be measured experimentally^52−55^ or estimated by parameter fitting of numerical simulations to experiments.^56,57^ Steen^58,59^ recently used driven droplet oscillations to estimate the magnitude of the contact line friction coefficient. The values of the line friction parameter in previous studies are in the order of 0.1 Pa·s for water and increase in proportion to the square root of the liquid viscosity up to ∼1 Pa·s.^53,56,60^ Since μ_f_ is significantly larger than liquid viscosity for most aqueous solutions,^52,56,60^ the contact line friction plays a particularly dominant role in dynamic and forced wetting applications. For example, for a spontaneous droplet spreading, modeling of the contact line without the contact line friction overestimates the spreading speed.^61,62^

The sensitivity of the line friction parameter to surface properties has been investigated thoroughly within the context of spontaneous spreading (i.e., zero impact speed). The relevant nondimensional number in liquid spreading is the line-friction Ohnesorge number ,(60) where ρ and *R*_0_ are the density and initial radius of the droplet, respectively. The line-friction Ohnesorge number quantifies the contribution of the line friction dissipation to the total kinetic energy.^60^ One may therefore expect that when *Oh*_f_ ≫ 1, the contact line speed is strongly influenced by the properties of the substrate and, in particular, the details of the surface geometry. In this surface-sensitive regime, Carlson et al.^56^ have shown that when the time is normalized with the time scale based on the line friction parameter, the initial rapid spreading of different droplets on smooth surfaces nearly collapses into one curve.

### Scope of the Present Study

For droplet impact on smooth surfaces, Wang et al.^63^ rescaled previous experimental data with contact line friction to demonstrate that line friction limits the maximum spreading radius β_max_. They suggested the scaling β_max_ ∼ (*Re*μ/μ_f_)^1/2^, where μ is the liquid viscosity and *Re* is the Reynolds number. However, to the best of our knowledge, no study has discussed the spreading resistance on microstructured surfaces based on the spreading mechanisms.

In our previous work,^64^ the spontaneous spreading of a droplet on hydrophilic slanted microstructures (see the inset in Figure 2a) was explained by mechanisms referred to as “slip”, “stick”, and “leap”. The spreading in the direction against the inclination (indicated by the red arrow in Figure 2a) was driven by the slip mechanism, i.e., a so-called “capillary spreading” driven by uncompensated Young’s force. In the direction with the inclination (indicated by the blue arrow in Figure 2a), the contact line motion could be explained by a combination of “slip”, “stick”, and “leap”; the contact line is pinned at the acute corner of the surface microstructures and the average spreading velocity is set by a combination of the capillary spreading on the flat fraction of the surface and “leaping” of the contact line to the next rise of the surface after the pinning. Here, we assume a length scale separation between the droplet size and the microstructures so that the spreading mechanisms can be considered local at the contact line. We also note that for hydrophobic asymmetric microstructures, pinning may occur in the direction against the inclination as well. Then, the spreading in both directions is expected to follow the same mechanisms: the combination of “stick” and “leap”.^64^ Therefore, the spreading can be symmetric for the hydrophobic asymmetric microstructures.

**Figure 1:**
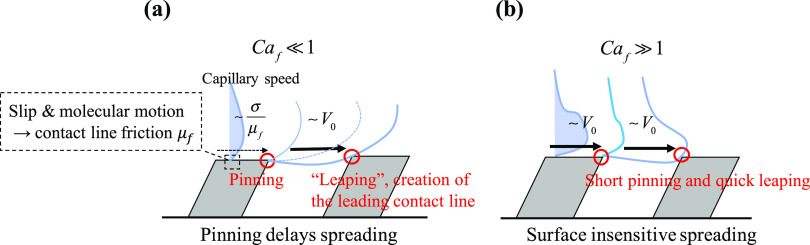
Schematics of two limits of droplet spreading on slanted ridges immediately after impact. (a) When *Ca*_f_ ≪ 1, line friction and pinning limit the spreading. (b) When *Ca*_f_ ≫ 1, fast leaping between ridges results in a surface-insensitive spreading.

In this work, we investigate the same microstructured surface as studied in ref (64), but now for impacting drops, which introduces the impact velocity *V*_0_ as an additional parameter. Here, we postulate that the impact velocity *V*_0_ determines the characteristic speed of “leaping”. The line friction parameter allows us to define the characteristic velocity of “slip” on the tip of the structures as σ/μ_f_ for a relatively small impact speed. This leads to a new measure of the spreading delay by the surface structures that consists of the ratio between *V*_0_ and the characteristic velocity σ/μ_f_. When *V*_0_ is small compared to σ/μ_f_, the contact line motion is significantly influenced by both pinning and line friction since the “leaping” between the structures takes longer than the “slip” on the top of the structures (see Figure 1a). In this situation, we expect the spreading to be hindered by the presence of the microstructures. On the other hand, a large impact speed results in a fast “leap” of the contact line to the next ridge, which effectively means that the underlying microscopic features of the surface geometry have a small influence on droplet impact (Figure 1b). We, therefore, propose that the line-friction capillary number, *Ca*_f_ = μ_f_*V*_0_/σ, is the relevant nondimensional number to characterize the influence of asymmetric surface geometry on droplet impact. For *Ca*_f_ ≪ 1, the spreading is delayed by the pinning on the asymmetric surface structures, and it is expected to be asymmetric (Figure 1a). Contrarily, the spreading is insensitive to the spreading mechanisms on the asymmetric surface and it would therefore be symmetric for *Ca*_f_ ≫ 1 (Figure 1b).

Note that despite the fact that the impact of a spherical drop on two-dimensional ridges is a three-dimensional problem, this study focuses on the local two-dimensional spreading across the asymmetric ridges. This can be motivated by the fact that the local curvature of the liquid–vapor interface near the spreading front in the cross-sectional plane is much smaller than the curvature in the horizontal plane.

## Materials and Methods

### Experimental Setup

**Figure 2:**
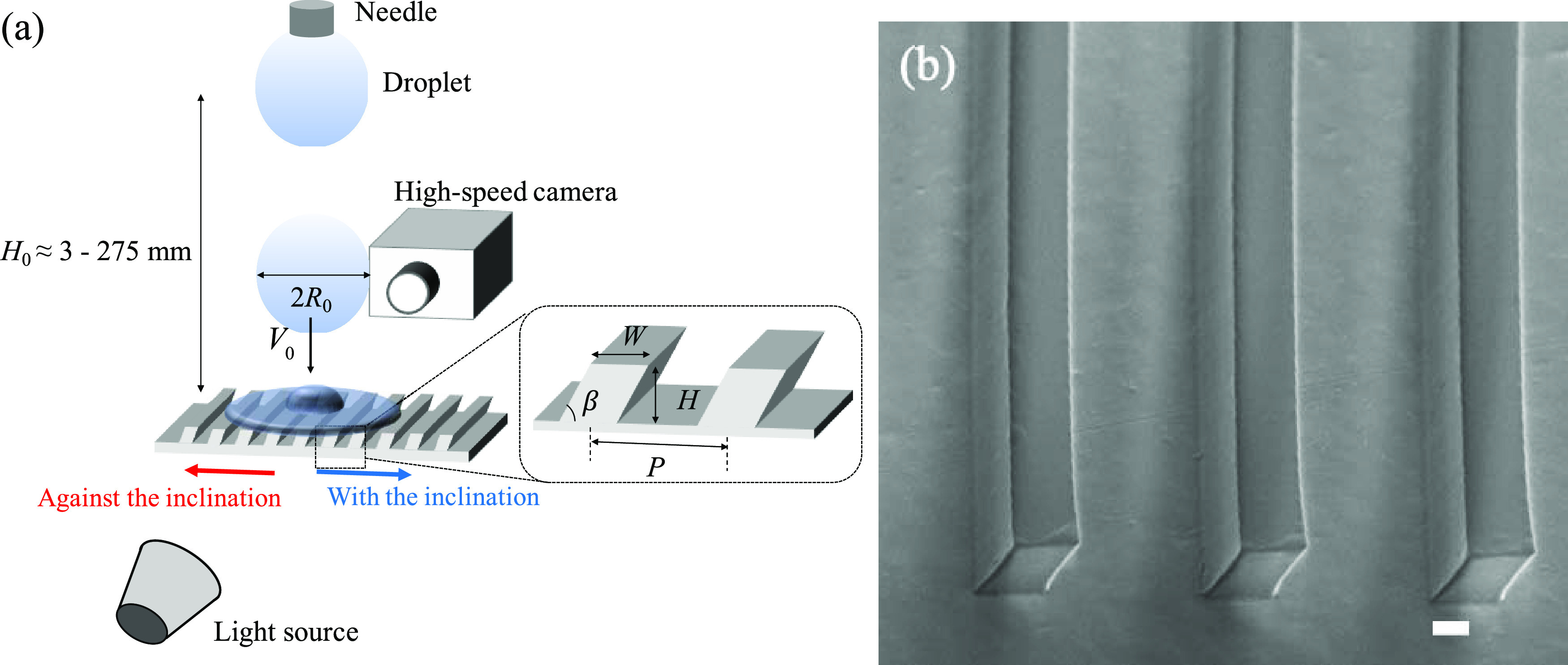
(a) Schematic of the droplet impact experiment. (b) Scanning electron microscopy image of the inclined microstructures for *P* = 60 μm. The scale bar indicates 10 μm.

Impact sequences of liquid droplets are observed with a high-speed camera (Dantec Speedsense M) at a frame rate of 8000 s^–1^ with spatial resolution of 15 μm. A schematic of the experimental setup is shown in Figure 2a. A liquid droplet is formed on the tip of a needle with an outer diameter of 0.31 mm (Hamilton, Gauge 30, point style 3) at a height *H*_0_ from the surfaces. The liquid is pumped by a syringe pump (Cetoni, neMESYS 1000N) at a small flow rate (0.10 μL/s). When the growing droplet has reached a certain radius, it pinches off from the needle and is accelerated by gravity and hits the substrate with an impact velocity *V*_0_. The impact velocities, which are varied by changing the distance from the substrate to the needle *H*_0_, are estimated from images before the droplet makes contact with the substrate. The height *H*_0_ is varied from 3 to 275 mm, which leads to impact velocities from 0.16 to 2.3 m/s (Table 1). Spontaneous spreading corresponding to *V*_0_ = 0 m/s is also measured. Fluid properties were varied by mixing deionized water, ethanol, and glycerol to change viscosity and surface tension. We label mixtures of water, glycerol, and ethanol (weight ratio of 1:2:1) and water and glycerol (weight ratio of 1:2) as “aq. glycerol–ethanol” and “aq. glycerol”, respectively. Fluid properties are shown in Table 2. The density of the liquids is estimated based on the mass fraction, using the literature values.^65,66^ Viscosity and surface tension are measured with a viscometer (Brookfield) and a TD 2 tensiometer (LAUDA), respectively.

**Table 1:** Height, *H*_0_, Impact Velocity, *V*_0_, and Friction Capillary Number, *Ca*_f_ = μ_f_*V*_0_/σ

*H*_0_ (mm)	3	5	10	25	40	135	275
*V*_0_ (m/s)	0.16	0.25	0.37	0.69	0.87	1.6	2.3
*Ca*_f_ for water	0.27	0.42	0.62	1.2	1.5	2.7	3.8
*Ca*_f_ for aq. glycerol–ethanol	0.67	1.0	1.5	2.9	3.6	6.7	9.6
*Ca*_f_ for aq. glycerol	0.89	1.4	2.1	3.8	4.8	8.9	12.8

**Table 2:** Liquid Properties: Density, ρ; Dynamic Viscosity, μ; Surface Tension, σ; Initial Radius, *R*_0_; Static, Advancing, and Receding Contact Angles on a Flat Surface, θ_e_, θ_a_, and θ_r_, Respectively; Line Friction Parameter, μ_f_; and Capillary Spreading Velocity, σ/μ_f_

label	ρ (kg/m^3^)	μ (mPa·s)	σ (mN/m)	*R*_0_ (mm)	θ_e_ (deg)	θ_a_ (deg)	θ_r_ (deg)	μ_f_ (Pa·s)	σ/μ_f_ (m/s)
water	997	0.992	72	1.1	50	70	27	0.12	0.60
aq. glycerol–ethanol	1075	11.7	34	0.9	34	59	22	0.14	0.24
aq. glycerol	1172	15.7	63	1.0	54	66	28	0.36	0.18

### Surface Preparation

The substrates studied are made from Ostemer 220 (Mercene Labs), a UV-curing Off-Stoichiometry-Thiol-Ene (OSTE) resin.^67^ The resin enables us to fabricate inclined micropatterns by exposing UV light at an oblique angle. The surfaces are prepared in three steps. First, a base OSTE layer is prepared on a smooth plastic film. Second, inclined microridges are patterned on the base OSTE layer by exposing ultraviolet light through a patterned mask. Finally, after cleaning uncured OSTE in an acetone bath, hydrophilic surface modification using 1% hydroxylated methacrylate (2-hydroxyethyl methacrylate, Sigma-Aldrich) solution in isopropanol with 0.05% benzophenone (Sigma-Aldrich) initiator is performed to achieve partial wetting so that the static contact angle on a flat surface is 50° for deionized water. Advancing and receding contact angles are measured with the sessile drop method.^68^ A sessile droplet with the initial volume of 5 μL is deposited on the surface, and it is pumped and drained by the syringe pump with a flow rate of 0.1 μL/s to measure advancing and receding contact angles, respectively. The contact angle right before the contact line starts to advance (recede) is defined as the advancing (receding) contact angle. The inclination of the ridges β is 60° (see Supporting Information Figure S1). Surface structures are characterized with scanning electron microscopy, and the width *W* and height *H* are 20 and 20 μm, respectively, as shown in Figure 2b. Two types of textures with *P* = 30 μm and *P* = 60 μm are investigated.

To determine the line friction parameter, experiments of a droplet spreading on a flat surface are modeled numerically^56^ (see Figure S1a in the Supporting Information). The line friction parameter is determined by fitting the spreading curve with the experiments. The spreading of a droplet on a flat surface is experimentally observed with a high-speed camera and the spreading radius and the spreading time are recorded. The detailed procedures to estimate the line friction parameter and the numerical details are available in the Supporting Information. The fitted line friction parameters are shown in Table 2.

## Results

### Comparison between Flat and Microstructured Surfaces

**Figure 3:**
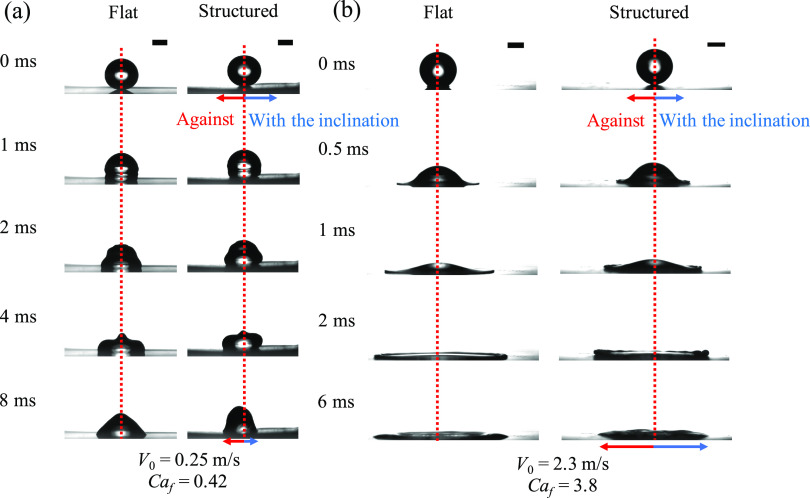
Selected captured images of experimental observations for (a) *V*_0_ = 0.25 m/s and (b) *V*_0_ = 2.3 m/s of a water droplet. Scale bars represent 1 mm. The structured surfaces here are with *P* = 60 μm.

Figure 3a shows a series of images of a water droplet spreading after impact on flat and asymmetrically microstructured surfaces with *V*_0_ = 0.25 m/s (*Ca*_f_ = 0.42). We observe that the droplet spreads not only slower on the asymmetric structures compared to the flat surface but also asymmetrically (Figure 3a). Specifically, the spreading is faster in the direction against the inclination of the ridge than in the direction with the inclination.

**Figure 4:**
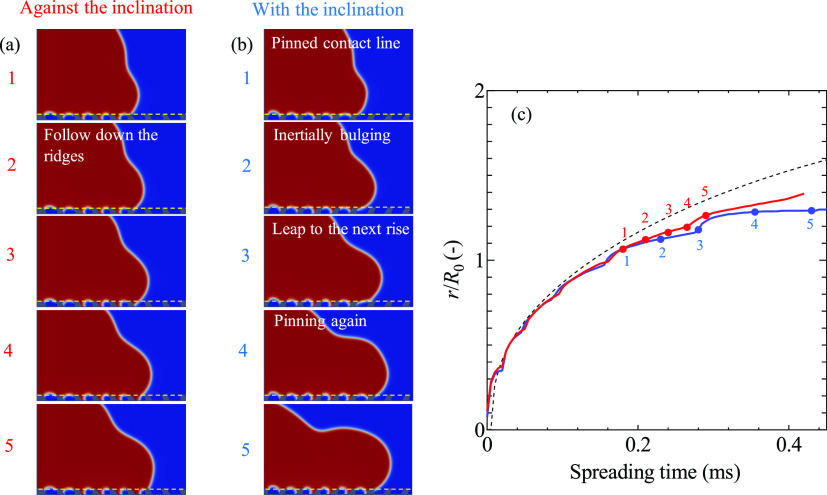
Spreading mechanisms of a water droplet after impact on the inclined microstructures with *V*_0_ = 0.8 m/s (*Ca*_f_ = 1.3). The pitch between the ridges *P* is 60 μm. (a) In the direction against the inclination, the contact line follows the surface structures. (b) In the direction with the inclination, the contact line is pinned at the acute corner of the surface (1, 4). Eventually, the liquid–vapor interface reaches to the next rise of the surface (2, 3). (c) Simulated spreading radius with respect to time on the flat surface (black dash line) and in the direction against (red line) and with (blue line) the inclination on the asymmetric microstructure. The red and blue points 1–5 correspond to the images in (a) and (b). The yellow dashed lines in (a, b) show the horizontal line where the spreading radius is measured.

Here, the numerical simulations of a water droplet impacting on the asymmetric microstructures with *V*_0_ = 0.8 m/s shown in Figure 4 reveal the spreading mechanisms on the asymmetric microstructure. The details of the simulations are provided in the Supporting Information. Note that the radius of the droplet in the simulation is reduced to 0.3 mm for computational costs. In the direction against the inclination, the contact line follows along the microstructure without pinning. As a consequence, it travels a longer path compared to its flat counterpart and therefore the apparent spreading rate is slightly slower (Figure 4a). In the direction with the inclination, the contact line spreads only on the tip of the surface ridges before it is temporarily pinned at the acute corner of the surface (1, Figure 4b). During pinning, the liquid–air interface is stretched until it reaches the next rise of the surface (2–3, Figure 4b). The spreading in this direction is delayed by the surface geometry compared to the flat surface if the duration of the pinning is longer than the time it would take for the interface to spread over a flat surface. Note that this mechanism is very similar to the slipping mechanism of a droplet on superhydrophobic surfaces observed experimentally with laser scanning confocal microscopy.^69^ At *Ca*_f_ = 1.3, a slight spreading asymmetry is observed in the simulation as shown in Figure 4c. Also note that the simulations are carried out in axisymmetric geometries so the structures in the simulations have a ring-like shape, slightly different from straight ridges in the experiments. The numerical model therefore only provides a qualitative picture of the asymmetric spreading. In the experiments, the cavity between the ridges might be filled up with the liquid phase immediately due to three-dimensional effects. However, we do not observe such filling in the experiment due to the lack of spatial resolution. The influence of such filling on the spreading is expected to be limited since the surface energy of the liquid–vapor interface between the ridges is smaller than the kinetic energy of the droplet.

Figure 3b shows snapshots of a droplet with *V*_0_ = 2.3 m/s (*Ca*_f_ = 3.8) on flat and asymmetric surfaces. We observe symmetric spreading on the microstructured surface, indicating a small effect of microstructured geometry on liquid spreading. In this case, the impact velocity reduces the pinning time and favors the leaping mechanism (Figure 1b).

**Figure 5:**
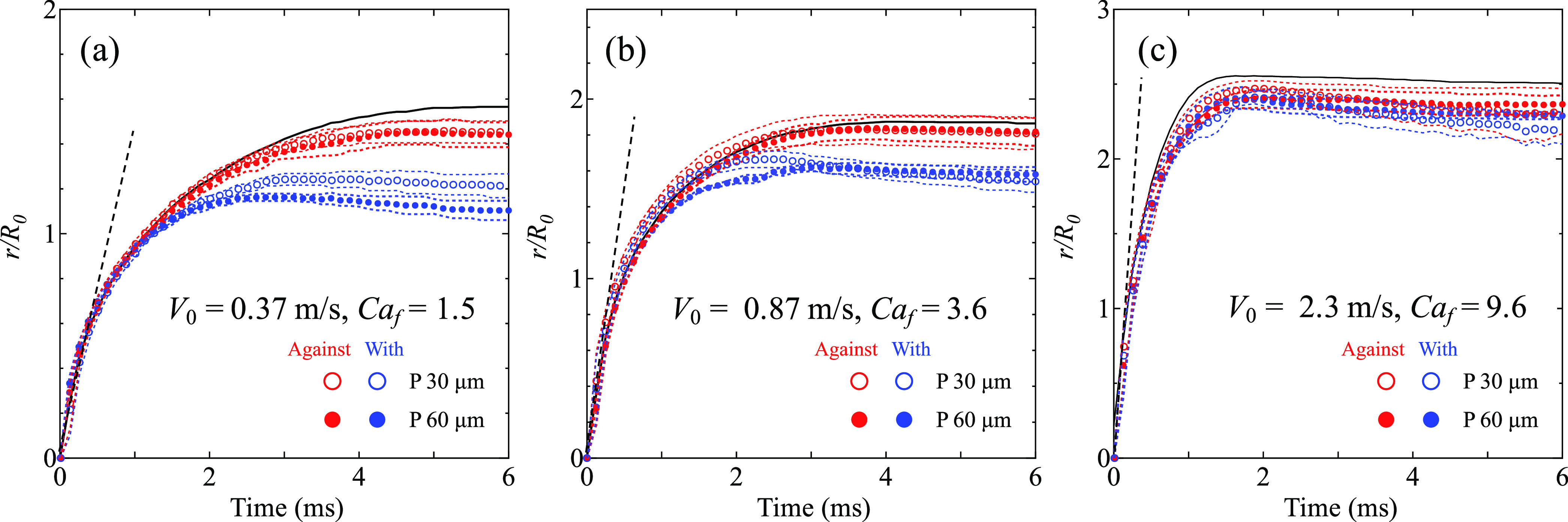
Experimentally observed spreading radius *r*/*R*_0_ of a droplet of aq. glycerol–ethanol as a function of time for *V*_0_ (a) 0.37 m/s, (b) 0.87 m/s, and (c) 2.3 m/s. Hollow markers represent surfaces with *P* = 30 μm and filled markers represent *P* = 60 μm. Black curves represent flat surface. Dash lines show the initial slope of the spreading curves. The data are averages of at least four repeated measurements. Dotted lines represent the standard deviations.

Figure 5 shows the spreading curves of droplets after impact of aq. glycerol–ethanol with three different impact velocities. In all three cases in Figure 5a–c, the spreading curves on the flat surface and asymmetric microstructures collapse in the initial phase, until around 1 ms. The spreading velocity in this phase—estimated from the slope of the spreading curve in the initial phase—is significantly higher than the impact velocity. For example, in Figure 5a, it is ∼1 m/s, which is a factor of 4 faster than the impact velocity. The spreading in this very initial phase is fully inertial and essentially independent of the contact line friction and consequently also insensitive to the surface structures. After the initial phase, the spreading curves in the direction against and with the inclination begin to deviate from each other (Figure 5a,b). Specifically, the spreading in the direction against the inclination (red markers) closely follows the one of the flat surface (black curves). In this direction, the small reduction in spreading velocity can be attributed to the increase of the wetted area of the microstructured surface compared to the flat surface and not to different spreading mechanisms. On the other hand, the spreading in the direction with the inclination (blue markers) is slowed down significantly. At these low impact velocities, this can be attributed to the pinning of the contact line at the acute corner of the structures. Moreover, we observe the subtle influence of the pitch on the spreading. The droplet spreads similarly on the surfaces with *P* = 30 μm and *P* = 60 μm.

In contrast, in Figure 5c, for a high impact velocity, the spreading curve in the direction with the inclination approaches the curve of the flat surface. Here, the pinning time becomes shorter and the delay by the surface structure in the direction with the inclination diminishes, as could be expected by *Ca*_f_ ≫ 1. Consequently, the spreading is nearly symmetric on the asymmetric microstructure over the entire spreading and close to the spreading on the flat surface.

### Maximum Spreading Radius

**Figure 6:**
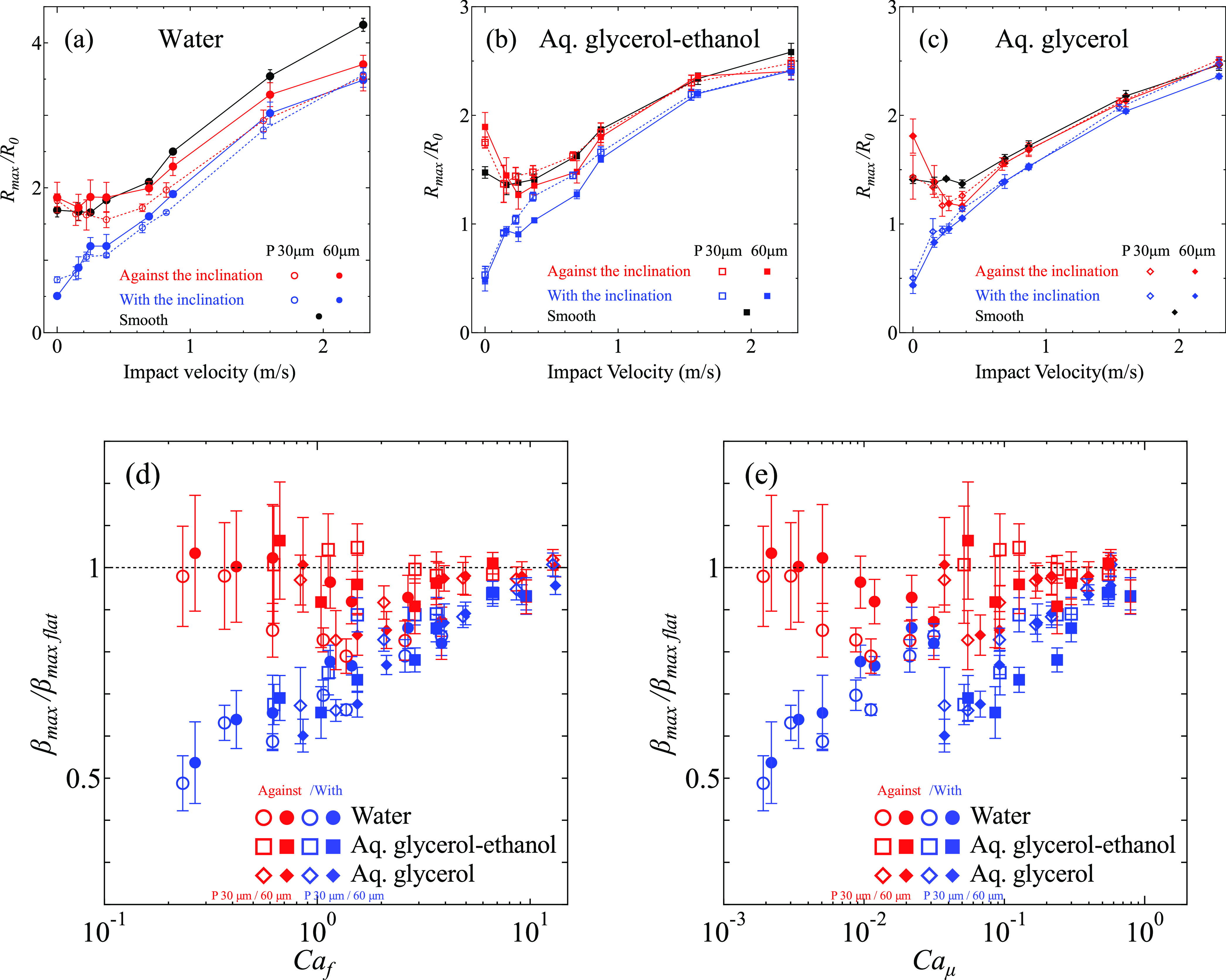
(a–c) Normalized maximum spreading radius with respect to *V*_0_ of (a) water, (b) aq. glycerol–ethanol, and (c) aq. glycerol. Black, red, and blue marks represent flat surfaces, the direction against the inclination, and with the inclination on the asymmetric microstructures, respectively. Hollow markers represent surfaces with *P* = 30 μm, and filled markers represent *P* = 60 μm. (d, e) Relative spreading factor to the flat surface with respect to (d) *Ca*_f_ and (e) *Ca*_μ_. The spontaneous spreading cases (*Ca*_f_ = 0) are eliminated in (d, e). Error bars in (a–e) indicate standard deviations.

Figure 6a–c shows the normalized maximum spreading radius, the so-called “spreading factor”, β_max_ = *R*_max_/*R*_0_, with respect to the impact velocity. At a low impact velocity, the maximum spreading on flat surfaces (black curves) is relatively independent of the impact velocity. This implies that the spreading after the impact at this velocity is similar to the spontaneous spreading of a deposited droplet (*V*_0_ = 0 m/s). The spreading factor increases with impact velocity above *V*_0_ ∼ 1 m/s, as the spreading gradually becomes more dominated by the impact. On asymmetric microstructured surfaces, the spreading factor in the direction against the inclination (red curve) follows the spreading factor on the flat surface, except for the water droplet with high impact velocity (Figure 6a). In contrast, the spreading factor in the direction with the inclination (blue curve) is smaller than the flat surface, but it approaches that of the flat surface as the impact velocity increases. The reduced pinning time with the increased impact velocity is responsible for this trend. We note the maximum radius in the direction against the inclination at *V*_0_ = 0 m/s is larger than at *V*_0_ = 0.16 m/s since the equilibrium position of the droplet is largely displaced to the direction against the inclination. The spreading in the direction with the inclination is significantly hindered at *V*_0_ = 0 m/s. The entire droplet is therefore displaced to the direction against the inclination.

Figure 6d shows the spreading factor on the asymmetric microstructured surface normalized by the spreading factor on the flat surface with the same impact velocity. The horizontal axis shows the line-friction capillary number. The normalized spreading factor in the direction against the inclination is almost constant around 1. Meanwhile, the normalized spreading factor in the direction with the inclination monotonically increases from 0.5 to 1 with increasing *Ca*_f_. As a result, the asymmetry in the spreading factor decreases monotonically with increasing *Ca*_f_, while for *V*_0_ = 0 m/s, the spreading factor in the direction against the inclination is a factor of 3 larger than in the direction with the inclination (see Figure 6a–c).

It is noticeable that the data for *P* = 30 μm and *P* = 60 μm follow the same trend. It is also important to note that the conventional capillary number *Ca*_μ_ = μ*V*_0_/σ does not give a monotonic trend (Figure 6e).

Here, the influence of viscosity and surface tension on the spreading asymmetry is considered through the friction capillary number. Liquid viscosity influences the line friction as μ_f_ ∝ μ^1/2^ for water–glycerol mixtures.^53,56,60^ Therefore, more viscous fluids are likely to have higher *Ca*_f_ for the same impact velocity, i.e., less sensitive to the surface structure. Similarly, a liquid with low surface tension is likely to be insensitive to asymmetric surface structures. In particular, for a very viscous liquid μ ≫ 1 Pa·s, the line friction parameter is possibly smaller than viscosity although it has not been seen in the experiments. In this situation, the viscous effect is more dominant than the line friction and the viscous and inertial effects would govern the behavior of the droplet.

**Figure 7:**
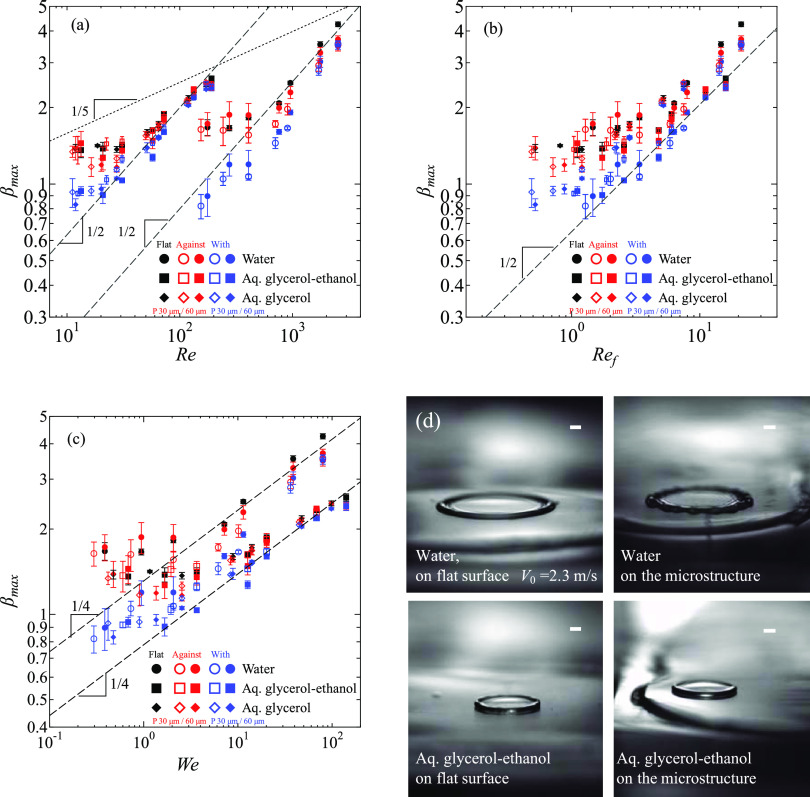
(a–c) Normalized maximum spreading radius with respect to (a) Reynolds number *Re* = ρ*R*_0_*V*_0_/μ, (b) Reynolds number based on the line friction parameter *Re*_f_ = ρ*R*_0_*V*_0_/μ_f_, and (c) Weber number *We* = ρ*R*_0_*V*_0_^2^/σ. Hollow markers represent surfaces with *P* = 30 μm, and filled markers represent *P* = 60 μm. Error bars in (a–c) indicate standard deviations. (d) Liquid lamella of water (top) and aq. glycerol–ethanol (bottom) on the flat surface (left) and the microstructured surfaces (right) at the moment of the maximum spreading radius with *V*_0_ = 2.3 m/s. The images are taken with an oblique angle. Scale bars indicate 1 mm.

Figure 7a show the spreading factor with respect to Reynolds number *Re* = ρ*V*_0_*R*_0_/μ. The well-known relation with the Reynolds number
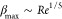
2is theoretically derived assuming that the kinetic energy is solely dissipated by viscous dissipation.^19,22,27^ Note that this is valid only in the viscous regime (i.e., for low Reynolds number). However, the spreading factors in our study do not follow eq 2, but β_max_ ∼ *Re*^1/2^, as seen in Figure 7a. This also agrees well with previous experimental observations of Lin et al.^27^ for high Reynolds numbers. Using an energy balance analysis, Wang et al.^63^ proposed the following scaling of the spreading factor
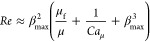
3The three terms represent the contributions to the energy budget by the contact line dissipation, work done by surface tension, and viscous dissipation, respectively. Here, the first term is the leading term in eq 3 in our study, i.e., μ_f_/μ ≫ 1/*Ca*_μ_ + β_max_^3^, and we obtain
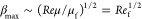
4where *Re*_f_ = ρ*R*_0_*V*_0_/μ_f_ is the Reynolds number based on the friction parameter. Note that the exponent in eq 4 agrees with our experiments (see Figure 7a). For more viscous fluids, when β_max_^3^ ≫ μ_f_/μ + 1/*Ca*_μ_, the classical scaling law for the viscous regime (eq 2) is recovered. In Figure 7b, the maximum spreading radius is plotted with *Re*_f_. The data follow β_max_ ∼ *Re*_f_^1/2^ for high *Re*_f_ and each direction follows each distinctive trend for low *Re*_f_.

Meanwhile, the well-known relation between the spreading factor and Weber number is^22,27^
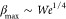
5in the capillary regime with low Ohnesorge number, while a lower exponent (1/6) is reported for viscous fluids with *Oh* = 0.585.^17,22^ An analysis based on the momentum and mass conservation leads to eq 5.^22^ The spreading factor in this work follows eq 5 well (Figure 7c). This is in reasonable agreement with previous studies^22,27^ since *Oh* in this study ranges from 3.6 × 10^–3^ to 6.4 × 10^–2^, which is regarded as the capillary regime. To conclude the scaling analysis, our experimental parameter space is in the capillary regime and the classical scaling law with Weber number is observed. The classical scaling with the Reynolds number (eq 2) must be reconsidered in the capillary regime, and the scaling (eq 4) is theoretically obtained by applying the energy balance analysis by Wang et al.^63^

The spreading factor on the microstructures for high *Re* (water, *V*_0_ = 2.3 m/s) does not reach the value on the flat surfaces since the liquid lamella begins to break earlier on the microstructured surfaces compared to flat surfaces. As shown in Figure 7d, the water lamella breaks only on the microstructures but not on the flat surface. On the other hand, the lamella of aq. glycerol–ethanol is stable at *V*_0_ = 2.3 m/s both on the flat and the microstructured surfaces. This can be understood as the instability of the wetting front leading to a splash. A criterion for splash is .^2,7^ For the water droplet with *V*_0_ = 2.3 m/s, we obtain *K* ∼ 4000, which is higher than the critical *K*, while *K* ∼ 2000 for the aq. glycerol–ethanol. Therefore, the instability of the water lamella in Figure 7d can be understood as the onset of a splash induced by the surface structure. It is responsible for the smaller spreading factor on the microstructured surface compared to the flat surface of a water droplet for a high impact velocity.

In practical situations such as raindrops^70^ and inkjet printing,^71,72^ the impact velocities can be beyond the velocity we investigate, as high as 10 m/s for raindrops, for example. In such situations, *Ca*_f_ ≫ 1 is expected and the spreading is insensitive to the organized microstructures. This implies that the microstructures are not very effective to harness such highly inertial droplets.

## Conclusions

Spreading of a droplet after impact on asymmetrically microstructured surfaces has been experimentally investigated. Considering the microscopic spreading mechanisms, the line-friction capillary number *Ca*_f_ = μ_f_*V*_0_/σ is proposed to distinguish between symmetric and asymmetric droplet spreading after impact. This nondimensional number describes the ratio between the impact velocity and the capillary speed. For the tilted microscale ridges considered here, the spreading in the direction against the inclination is not very sensitive to the surface structures, while the spreading in the direction with the inclination scales well with *Ca*_f_. Consequently, the asymmetry in the maximum spreading radius fades out with increasing *Ca*_f_. The scaling law for the spreading factor with Weber number (β_max_ ∼ *We*^1/4^) is confirmed to hold for spreading on asymmetric surfaces. However, the scaling law with Reynolds number shows a larger exponent than in the classical theories (β_max_ ∼ *Re*^1/5^). The spreading factor in our experiments follows the scaling proposed by Wang et al.,^63^ which takes the energy dissipation at the contact line into account in the energy balance analysis. Further work considering other surface geometries such as inclined cones and posts is needed to see if *Ca*_f_ ≲ 1 can be used as a general condition to distinguish between symmetric and asymmetric spreading after droplet impact. Especially, the spanwise density of such structures can be crucial since the influence of the pinning depends on it. The critical capillary number above which the spreading asymmetry diminishes may be dependent on the spanwise density.

## Supporting Information Available

Cross-sectional scanning electron microscopy image of the microstructured surface and detailed procedures to determine the line friction parameter and the numerical details (PDF)
Video animation of a simulated droplet impact spreading in the direction against the inclination (MP4)
Video animation of a simulated droplet impact spreading in the direction with the inclination (MP4)
