# Supplementary material for: Droplet Impact on Surfaces with Asymmetric Microscopic Features
Source: Langmuir. 2021 Sep 1;37(36):10849–58. doi: 10.1021/acs.langmuir.1c01813 (PMC8447403; doi:10.1021/acs.langmuir.1c01813)
Supplement: Supplementary file 1 — la1c01813_si_001.pdf [file la1c01813_si_001.pdf]

Supporting Information: Droplet impact on surfaces with asymmetric microscopic features

Susumu Yada,*,† Blandine Allais,‡ Wouter van der Wijngaart,¶ Fredrik Lundell,† Gustav Amberg,†,§ and Shervin Bagheri†

†*Department of Engineering Mechanics, Royal Institute of Technology, 100 44 Stockholm, Sweden*

‡*École Normale Supérieure de Lyon, 69342 Lyon, France*

¶ *Division of Micro and Nanosystems, Royal Institute of Technology, 100 44 Stockholm, Sweden*

§*Södertörn University, 141 89 Stockholm, Sweden*

E-mail: susumuy@mech.kth.se

Table of contents

Procedure to estimate the line friction parameter **S2**

Numerical simulation of droplet impact **S4**

Figure S1. Cross sectional scanning electron microscopy image of the microstructures **S5**

Figure S2. The spreading experiments and the numerical simulations to estimate the line friction parameter **S5**

References **S6**

Procedure to estimate the line friction parameter

Experiments of a droplet spreading on a flat surface are modelled numerically to determine the line friction parameter. The line friction parameter is determined by fitting the spreading curve with the experiments. Spreading of a droplet on a flat surface is experimentally observed with a high-speed camera at a frame rate of 52000 s^{-1} and the spreading radius and the spreading time are recorded. The spreading experiments and the numerical simulations for water are shown in Fig. S2(a). To enhance sensitivity to the line friction parameter,[S1] we reduce the initial radius to 0.4 mm.

Navier-Stokes-Cahn-Hilliard equations are solved using in-house software "FemLego" to obtain the spreading radius for different values of μ_f. FemLego is an adaptive finite element toolbox where weak formulation of partial differential equations is defined on a MAPLE worksheet.[S2] The numerical model is composed of the Navier-Stokes equations and the Cahn-Hilliard equation;

$$\rho(c)\frac{D\boldsymbol{u}}{Dt} = -\frac{1}{Re}\nabla p + \frac{1}{Re}\nabla \cdot \mu(c)(\nabla \boldsymbol{u} + \nabla^T \boldsymbol{u}) - \frac{c\nabla\phi(c)}{Ca_\mu \cdot Cn \cdot Re}, \tag{S1}$$

$$\nabla \cdot \boldsymbol{u} = 0, \tag{S2}$$

$$\frac{Dc}{Dt} = \frac{1}{Pe}\nabla^2\phi(c). \tag{S3}$$

The Navier-Stokes equations are characterized by the capillary number $Ca_\mu{=}\mu U/\sigma$, the Reynolds number $Re{=}\rho UL/\mu$, and the Cahn number $Cn{=}\epsilon/L$, where ρ and μ are the density and viscosity of the liquid phase, σ is the surface tension of the liquid-vapor interface, and ϵ is the diffuse interface width. Moreover, U and L are the characteristic velocity and length of the system, respectively. Here, capillary velocity σ/μ and the initial droplet radius R_0 are chosen as the characteristic velocity and length scales. The variable c is the phase field variable, where $c = 1$ represents the liquid phase, and $c = -1$ the vapor phase.

In the Cahn-Hilliard equation (S3), ϕ is the chemical potential of the system defined as

$\phi = \Psi'(c) - Cn\nabla^2 c$. Here, $\Psi(c) = (c+1)^2(c-1)^2/4$ is the double well function, where the minimum represents the stable phases for vapor ($c = -1$) and liquid ($c = 1$). The Peclet number is defined as $Pe = UL/D$ where D is a mass diffusivity.

The line friction parameter appears as a boundary condition in the Navier-Stokes-Cahn-Hilliard equations in the form[S3,S4]

$$-\epsilon\mu_f \frac{\partial c}{\partial t} = \epsilon\sigma\nabla c \cdot \boldsymbol{n} - \sigma\cos(\theta_e)g'(c), \tag{S4}$$

where θ_e is static contact angle. The polynomial $g(c) = 0.5 + 0.75c - 0.25c^3$ rapidly shifts from 0 (vapor phase $c = -1$) to 1 (liquid phase $c = 1$). The left hand side of Eq.S4 models the dissipation at the moving contact line.

Fluid properties in Table 2 are used in the simulations to match to the experiments. The mass diffusivity in Eq. S3 is fixed to 5.7 $\times 10^{-6}$ $m^2 s^{-1}$ for all of our simulations. The interface width ϵ is determined following the guidance to maintain the sharp interface limit.[S5] The simulations are carried out in the axi-symmetric geometry. The only unknown parameter in the numerical simulation is the line friction parameter. We impose the no-slip boundary condition at the wall for the velocity u and Eq. S4 as the wall boundary condition for the phase-field variable c. Therefore, the local effect at the contact line is effectively modeled by the line friction parameter in this work. The fitted spreading curves are shown in Fig. S2(b). The fitted line friction parameters are reported in Table 2. The line friction parameter increases with kinematic viscosity from 0.12 Pa·s (water) to 0.36 Pa·s (aq. glycerol).

Without the numerical simulations, the line friction parameter can be determined solely by experiments such as the droplet oscillatory measurement[S6,S7] and electrowetting experiments,[S8,S9] and the comparison of spreading experiments and molecular kinetics theory.[S10] Figure S2(c) shows the literature values of the line friction parameter of aqueous glycerol with air surrounding. The fitted friction parameters in this study are in reasonable agreement with the literature values with an exception of Ref. S10.

Numerical simulation of droplet impact

The droplet impact on the asymmetric microstructure is numerically modeled to investigate how the liquid-vapor interfaces proceed over the microstructures. The simulations reveal the spreading mechanisms over the microstructures, which can not be observed in the experiments due to lack of spatial and time resolution. Numerical simulations are performed to discuss the spreading mechanisms on the structured surfaces qualitatively, not to make a full comparison with the experiments.

In cylindrical coordinates, Eqs. (S1-S3) with Eq. S4 are solved with the properties of a water droplet in Table 2 including the fitted line friction parameter. To reduce the computational cost, the initial radius of the droplet is reduced to 0.3 mm, while the dimension of the surface geometry is identical to the experiment ($P = 60\mu m$). The droplet is initialized at the distance of $0.033R_0$ from the solid wall with the initial vertical velocity of 0.8 m/s. Video animations are available as Supplemental Material.

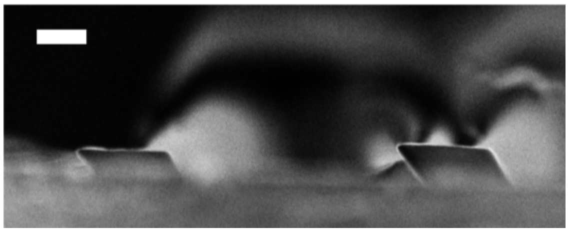

Figure S1: Cross sectional scanning electron microscopy image of the microstructured surface (W= 20 μm, P = 60 μm). The scale bar indicate 10 μm.

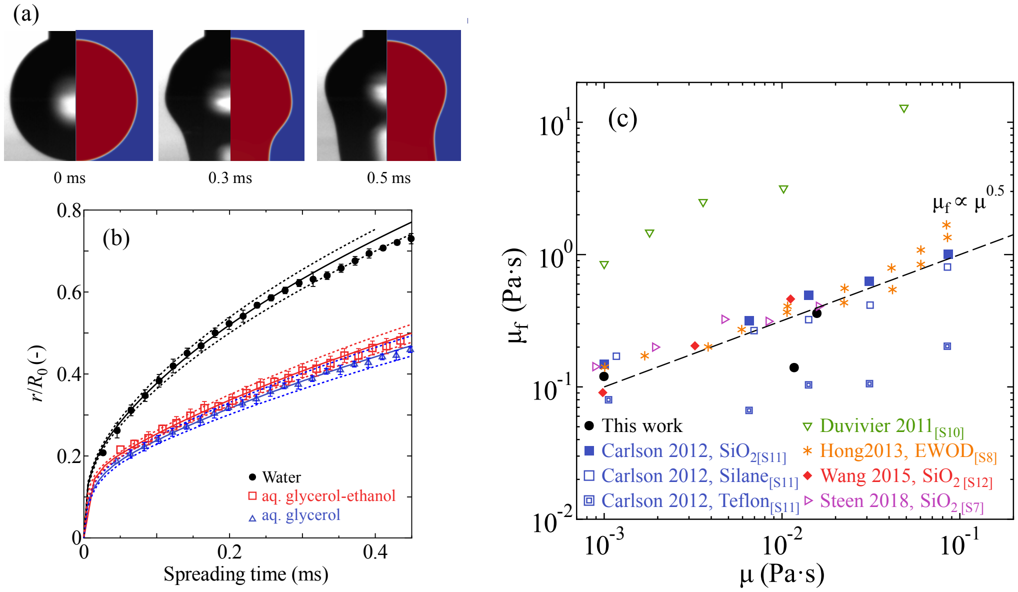

Figure S2: (a) The spreading experiments and the numerical simulations of a water droplet. (b) Spreading curves of spontaneous spreading droplets. The numerical curves (solid lines) are fitted to the experimental spreading curves in order to estimate the friction parameters shown in Table 2. The error bars indicate the standard deviations in the experiments. The dotted lines represent $\pm$20% deviations from the fitted line friction parameter. (c) Comparison of the line friction parameter of glycerol-water mixtures with respect to fluid viscosity. Ref. S11,S12 are measured with the fitting with the phase-field simulations to the droplet spreading experiments. Ref. S7 is measured with oscillatory droplet measurements. Ref. S10 is estimated with the comparison of droplet spreading experiments and molecular kinetics theory. Ref. S8 is measured with electrowetting on dielectric layers(EWOD). The outer fluid is air.

References

[S1] Do-Quang, M.; Shiomi, J.; Amberg, G. When and how surface structure determines the dynamics of partial wetting. *Eur. Phys. Lett.* **2015**, *110*, 46002.

[S2] Amberg, G.; Tönhardt, R.; Winkler, C. Finite element simulations using symbolic computing. *Math. Comp Simul.* **1999**, *49*, 257 – 274.

[S3] Jacqmin, D. Contact-line dynamics of a diffuse fluid interface. *J. Fluid Mech.* **2000**, *402*, 57–88.

[S4] Yue, P.; Feng, J. J. Wall energy relaxation in the Cahn–Hilliard model for moving contact lines. *Phys. Fluids* **2011**, *23*, 012106.

[S5] Yue, P.; Zhou, C.; Feng, J. J. Sharp-interface limit of the Cahn–Hilliard model for moving contact lines. *J. Fluid Mech.* **2010**, *645*, 279–294.

[S6] Xia, Y.; Steen, P. H. Moving contact-line mobility measured. *J. Fluid Mech.* **2018**, *841*, 767–783.

[S7] Xia, Y.; Steen, P. H. Dissipation of oscillatory contact lines using mode scanning. *npj Microgravity* **2020**, *6*, 3.

[S8] Hong, J.; Kim, Y. K.; Kang, K. H.; Oh, J. M.; Kang, I. S. Effects of Drop Size and Viscosity on Spreading Dynamics in DC Electrowetting. *Langmuir* **2013**, *29*, 9118–9125.

[S9] Vo, Q.; Tran, T. Contact line friction of electrowetting actuated viscous droplets. *Phys. Rev. E* **2018**, *97*, 063101.

[S10] Duvivier, D.; Seveno, D.; Rioboo, R.; Blake, T. D.; De Coninck, J. Experimental Evidence of the Role of Viscosity in the Molecular Kinetic Theory of Dynamic Wetting. *Langmuir* **2011**, *27*, 13015–13021.

[S11] Carlson, A.; Bellani, G.; Amberg, G. Universality in dynamic wetting dominated by contact-line friction. *Phys. Rev. E* **2012**, *85*, 045302(R).

[S12] Wang, J.; Do-Quang, M.; Cannon, J. J.; Yue, F.; Suzuki, Y.; Amberg, G.; Shiomi, J. Surface structure determines dynamic wetting. *Sci. Rep.* **2015**, *5*, 8474.
